# The Hull Fishermen's Hospital

**Published:** 1904-10-29

**Authors:** 


### THE HULL FISHERMEN'S HOSPITAL.

The *Alpha* hospital ship, to which some of the unfortunate victims of the North Sea disaster were taken and conveyed back to Hull, is a steam trawler belonging to the Royal National Mission to Deep Sea Fishermen. She is adequately fitted in every way, and carries an *x*-Rays equipment. The swing cots, which are particularly comfortable, are amidships—the steadiest part of a vessel. A specially constructed stretcher is used to carry the patient on to the *Alpha*, and once there his relief begins. The *Alpha*, like every other hospital ship belonging to the Mission to Deep Sea Fishermen, carries a fully qualified medical man, and every Mission skipper holds a certificate for "first aid." The ship's nurse is the surgeon's mate, who is also responsible for the care of the dispensary. The *Alpha's* dispensary is well administered, and does the surgeon's mate much credit. Besides being a floating hospital, the *Alpha* trawls, like any other steam trawler.

